# The bacterial microbiome in spider beetles and deathwatch beetles

**DOI:** 10.1128/spectrum.01981-24

**Published:** 2025-04-10

**Authors:** Austin Hendricks, T. Keith Philips, Tobias Engl, Rüdiger (Rudy) Plarre, Vincent G. Martinson

**Affiliations:** 1 Department of Biology, University of New Mexico 118833, Albuquerque, New Mexico, USA; 2 Department of Biology, Western Kentucky University 167037, Bowling Green, Kentucky, USA; 3 Department of Insect Symbiosis, Max Planck Institute for Chemical Ecology 28298, Jena, Thuringia, Germany; 4 Bundesanstalt für Materialforschung und -prüfung (BAM) 42220, Berlin, Berlin, Germany; China Agricultural University, Haidian, Beijing, China

**Keywords:** Ptinidae, Anobiidae, Dermestidae, Bostrichidae, Bostrichoidea, *Wolbachia*, *Sodalis*

## Abstract

The beetle family Ptinidae contains a number of economically important pests, such as the cigarette beetle *Lasioderma serricorne*, the drugstore beetle *Stegobium paniceum*, and the diverse spider beetles. Many of these species are stored product pests, which target a diverse range of food sources, from dried tobacco to books made with organic materials. Despite the threat that the 2,200 species of Ptinidae beetles pose, fewer than 50 have been surveyed for microbial symbionts, and only a handful have been screened using contemporary genomic methods. In this study, we screen 116 individual specimens that cover most subfamilies of Ptinidae, with outgroup beetles from closely related families Dermestidae, Endecatomidae, and Bostrichidae. We used 16S ribosomal RNA gene amplicon data to characterize the bacterial microbiomes of these specimens. The majority of these species had never been screened for microbes. We found that, unlike in their sister family, Bostrichidae, that has two mutualistic bacteria seen in most species, there are no consistent bacterial members of ptinid microbiomes. For specimens which had *Wolbachia* infections, we did additional screening using multilocus sequence typing and showed that our populations have different strains of *Wolbachia* than noted in previous publications.

**IMPORTANCE:**

Ptinid beetles are both household pests of pantry goods and economic pests of dried goods warehouses and cultural archives, such as libraries and museums. Currently, the most common pest control measures for ptinid beetles are phosphine and/or heat treatments. Many ptinid beetles have been observed to have increasing resistance to phosphine, and heat treatments are not appropriate for many of the goods commonly infested by ptinids. Pest control techniques focused on symbiotic bacteria have been shown to significantly decrease populations and often have the beneficial side effect of being more specific than other pest control techniques. This survey provides foundational information about the bacteria associated with diverse ptinid species, which may be used for future control efforts.

## INTRODUCTION

Many insects have expanded their niche space by developing close mutualisms with microbes. These relationships are generally split into two categories: facultative and obligate. While facultative symbionts provide diverse benefits of protection and nutrition, nearly all obligate symbionts provide nutritional benefits, but identifying the benefits provided by individual symbionts requires experimental manipulation. An alternative method to help predict their functional category can be observed by screening individuals. These symbiont types generally differ in their prevalence within and between host populations. Facultative symbionts can be either acquired from the parent (i.e., vertical inheritance) or from the environment (i.e., horizontal inheritance). While facultative mutualists provide benefits to the host under certain conditions (e.g., parasitoid pressure, heat stress), the cost to maintain the symbiont may leave hosts at a disadvantage when they no longer encounter the condition (1, 2). Therefore, facultative symbioses are not found in all populations of a host species or even in all individuals within a population (3). In contrast, obligate mutualists are found in all host individuals because both host and symbiont rely on the other for survival, and closely related hosts generally have closely related symbionts as a result of co-diversification. These characteristics differentiate obligate and facultative symbionts and can be used in surveys of novel taxa to identify potential symbiotic relationships.

Obligately mutualistic bacterial symbionts have evolved independently at least 16 times and are found in seven orders of insects: Blattodea, Coleoptera, Diptera, Hemiptera, Hymenoptera, Phthiraptera, and Psocoptera (4), but even more may be waiting to be identified. Beetles (Coleoptera) represent one of the most speciose groups of insects, with over 350,000 described species, rivaled only by Hymenoptera (5–7). Several beetle lineages are known to harbor microbial symbionts that include intracellular and extracellular bacteria and fungi (8).

**Fig 1:**
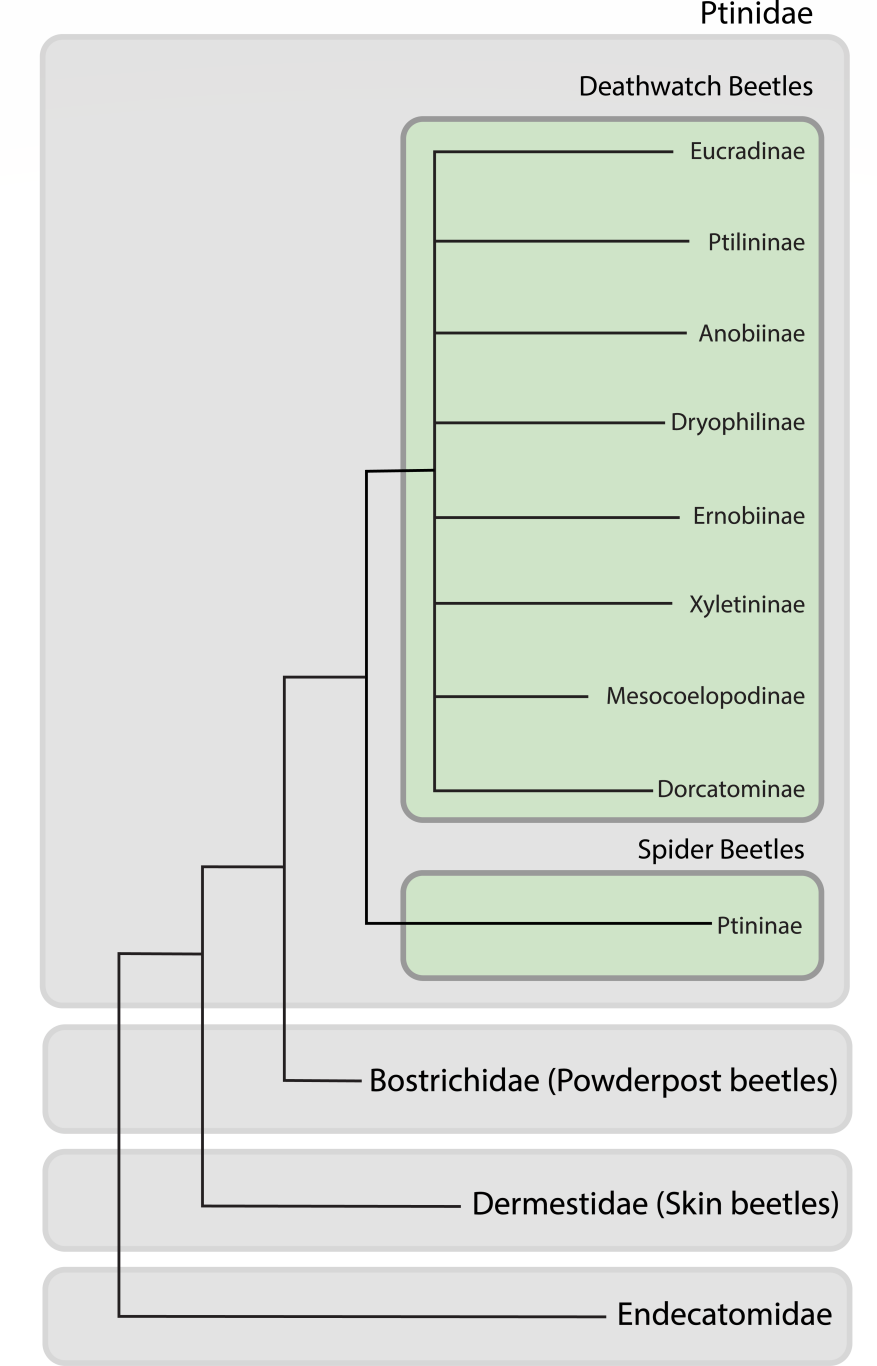
Dendrogram of Bostrichoid families and ptinid subfamilies. While the relationships among the families are not fully known, this figure is based on previously published phylogenies and dendrograms combining genetic, morphological, and dietary information.

One superfamily within Coleoptera that is responsible for a large number of timber and stored product pests is Bostrichoidea, which consists of the families Bostrichidae, Dermestidae, Ptinidae, and Endecatomidae (Fig. 1). Given the extensive number of beetle species we surveyed, there is also a correspondingly large variety of Latin nomenclature spanning multiple taxonomic levels, including superfamily, family, subfamily, genus, and species. To make this more accessible to a broader audience, we refer to family-level groups by a common name instead: Bostrichidae (powderpost beetles), Dermestidae (skin beetles), Anobiidae s.s. (deathwatch beetles), and Ptinidae s.s. (spider beetles). These lineages are specialists on difficult-to-digest, low-nutrient, and low-moisture substrates. While many survive on dead wood, others have become specialists in a wide range of human products, ranging from processed foods like flour or herbs to culturally important artifacts like books, textiles, wool carpets, clothing, and furniture, making them an issue in archives and libraries (9).

Bostrichoidea pests include *Rhyzopertha dominica* (lesser grain borer), which is primarily a pest of processed grain (10) but has also been observed to directly affect agricultural yields through infestation of olive trees (11), *Trogoderma granarium* (Khapra beetle), the world’s most destructive pest of stored grains and seeds, which is a quarantine species in Europe (12), and *Prostephanus truncatus* (larger grain borer), a major pest of maize that affects up to 30% of crops in Central America and Africa (13). Furthermore, powderpost beetles are notable because at least 28 species were recently found to harbor two bacterial endosymbionts: *Bostrichidicola* and *Shikimatogenerans* (14–16). These endosymbionts encode pathways for the synthesis of nutrients essential for cuticle development and hardening, which allows these stored product pests to persist in the extremely dry environments of product warehouses (15, 16). The remaining families of Bostrichoidea also contain specialized pests: skin beetles can survive in carpets and are used to strip skeletons of tissue; spider beetles are common pests of stored products and dried goods; and deathwatch beetles are mainly associated with wood but contain major dried product pests. Many of these species demonstrate similar resistance to desiccation as powderpost beetles (17); however, despite similarities in natural history and economic importance as pests, there have been no systematic studies for obligate endosymbionts that may aid them in desiccation resistance or nutrient supplementation.

Ptinidae comprises 230 genera and 2,200 species; however, there are likely many undocumented species because they are difficult to collect due to their small size and burrowing habits—their larvae are hidden inside dietary compounds (e.g., wood) (18). Broadly, the lineage can be split into two groups: the spider (subfamilies Ptininae and Gibbiinae) and deathwatch beetles (formerly Anobiidae; now the remaining nine subfamilies of Ptinidae; Fig. 1) (19, 20). While the most obvious difference between spider and deathwatch beetles is morphological—spider beetles resemble spiders—the spider beetles also have more varied diets beyond wood or stored products, with many being dung feeders or reliant on close associations with other insects and organisms (21). Although some deathwatch beetles were examined for endosymbionts by microscopy in the 1960s (22), no spider or deathwatch beetles have been confirmed to have bacterial endosymbionts. Furthermore, only five species of deathwatch beetles have been confirmed to have *fungal* nutritional endosymbionts: *Lasioderma serricorne* (cigarette beetle), *Stegobium paniceum* (drugstore beetle), *Xestobium plumbeum*, and at least two species in the genus *Ernobius* (23). Strikingly, these fungal endosymbionts are from distantly related subphyla (Saccharomycotina and Pezizomycotina—most recent common ancestor >500 Mya), indicating that symbioses with fungi have evolved independently multiple times (24). Moreover, these fungal symbionts may have been replacements for an ancestral bacterial symbiont related to that found in the common ancestor of all Bostrichoidea. Here, we screen 116 individual specimens representing 61 species of powderpost, skin, spider, and deathwatch beetles for possible bacterial endosymbionts using 16S ribosomal RNA gene amplicon sequencing. While amplicon sequencing is not able to differentiate between facultative and obligate endosymbionts or between endosymbionts and commensal/environmental microbes, we set out to (i) describe the taxonomic composition of ptinid beetle bacterial microbiomes and (ii) search for patterns in taxonomic composition that may indicate the presence of a highly conserved ancestral bacterial endosymbiont.

## MATERIALS AND METHODS

### Sampling

Specimens came from a variety of sources. All samples originated as individual adult beetles. For 71 specimens, we used previously extracted DNA (18, 25). These specimens were collected from a wide geographic range across four different continents. The remaining 55 samples were sourced from insect laboratory colonies. The spider beetles (*Gibbium aequinoctiale*, *Mezium affine*, *Mezium gracilicorne*, *Sphaericus gibbosus*) were sourced from the lab of TK Philips. The deathwatch beetle *Anobium punctatum* and the spider beetles *Gibbium psylloides* and *Niptus hololeucus* were sourced from the labs of T Engl and R Plarre. The deathwatch beetles *Lasioderma serricorne* and *Stegobium paniceum* each had one population sourced from the U.S. Department of Agriculture (USDA, E Scully, ARS, Plains Area, Manhattan, KS). The skin beetle *Dermestes maculatus* and additional *S. paniceum* and *L. serricorne* were sourced from colonies maintained at the University of New Mexico (Albuquerque, NM). Full sample information is available in Table S1. Whole beetles were processed by extracting DNA from individuals. Specimens were either preserved in 100% ethanol or freeze-dried. Ethanol-preserved specimens were dried for 1 h prior to processing in a sterile biosafety cabinet. Individual specimens were processed with the Qiagen DNeasy Blood & Tissue Kit (Qiagen, Hilden, Germany). Briefly, specimens were placed in RLT buffer and homogenized with a micro pestle, followed by further homogenization using a Qiagen TissueLyser for 3 min at 30 Hz with 100 µL of 0.1 mm Mini-BeadBeater zirconia–silicate beads (BioSpec Products, Oklahoma, USA). The remaining steps were done following the manufacturer’s protocol. Library preparation and sequencing were done at Novogene (Beijing, China) using primers 515F (GTGCCAGCMGCCGCGGTAA) and 806R (GGACTACHVGGGTWTCTAAT), which target the V4 region of the 16S ribosomal RNA gene. Of the original set of 126 samples, 116 passed the library preparation, representing 61 species. Sequencing was done on a 250 bp paired-end NovaSeq 6000 flow cell.

### Data QC and 16S rRNA analysis

Read counts ranged between 30,359 and 118,987, with a mean of 85,073 and a median of 90,263. Denoising was done through dada2 with a forward truncation length of 225 bp and a reverse truncation length of 224 bp. Amplicon sequence variants (ASVs) were generated by Qiime2 (26), and taxonomic designations were made by aligning these sequences to the Silva 138 database (27, 28). ASVs are an alternative to operational taxonomic units (OTUs), wherein each unique nucleotide sequence is counted, versus OTUs, which traditionally group sequences that meet certain similarity criteria (29). ASVs that had top alignments to either chloroplasts or eukaryotic mitochondria were removed from the data set. Percentages were generated based on the filtered data set and calculated simply based on the division of each ASV by the total number of reads for that sample (e.g., 1% of ASVs would mean there was one read for that ASV out of 100 total reads for one sample). Reads for this data set are available on SRA under BioProject ID PRJNA1167727. Bar plots were generated using the qiime2 R package (30). Species richness and diversity indices were calculated using the *vegan* R package (31). NMDS plots were also created using *vegan* and rarefied using the *rrarefy* function to 16,000 reads.

### Wolbachia typing

To characterize the *Wolbachia* strains identified by high-throughput sequencing, we performed additional PCR and sequencing on *L. serricorne* and *S. paniceum* samples focusing on five housekeeping genes commonly used for *Wolbachia* multilocus sequence typing (MLST): *hcpA*, *gatB*, *ftsZ*, *coxA*, and *fbpA* (32). Amplification was done with primers and cycling conditions utilized in previous studies (32, 33). Amplicons were sent for Sanger sequencing at Eurofins (Luxembourg City, Luxembourg) or for linear amplicon sequencing by Oxford Nanopore at Plasmidsaurus (Oregon, USA). Sequences were assigned an allele number through the PubMLST.org *Wolbachia* typing database, and GenBank IDs for our sequences can be found in Table 1 (34).

**TABLE 1:** Table of MLST gene classifications from each study population compared to examples from the literature

Publication	Population	gatB	coxA	hcpA	ftsZ	fbpA	wsp
*Lasioderma serricorne*							
Kageyama et al. (2010) (35)	Ls1	N/A	N/A	N/A	N/A	N/A	Single: Type 3 (16) AB469359
Li et al. (2015) (33)	Canada	4 KP762374	14 KP762359	40 KP762379	N/A	4 KP762366	N/A
Li et al. (2015) (33)	Spain	4	14	N/A	N/A	N/A	N/A
Li et al. (2015) (33)	Greece	96 KP762375	N/A	N/A	73 KP762386	N/A	N/A
This paper	UNM	N/A	14 PQ855642	N/A	N/A	4 PQ855654	N/A
This paper	USDA	4 PQ855639	14 PQ855643	40 PQ855646	243 PQ855649	444 PQ855652	N/A
This paper	Parsley	N/A	N/A	N/A	N/A	N/A	N/A
*Stegobium paniceum*							
Kageyama et al. (2010) (35)	Sp1	N/A	N/A	N/A	N/A	N/A	Double: types 13 (4) and 14 (9) AB469917 and AB469918
Li et al. (2015) (33)	USA	N/A	64 KP762360	N/A	63 KP762390	75 KP762367	N/A
This paper	UNM	103 PQ855640	64 PQ855644	40 PQ855647	243 PQ855650	444 PQ855653	N/A
This paper	USDA	103 PQ855641	64 PQ855645	40 PQ855648	243 PQ855651	N/A	N/A

## RESULTS AND DISCUSSION

### Overall microbiome diversity

**Fig 2:**
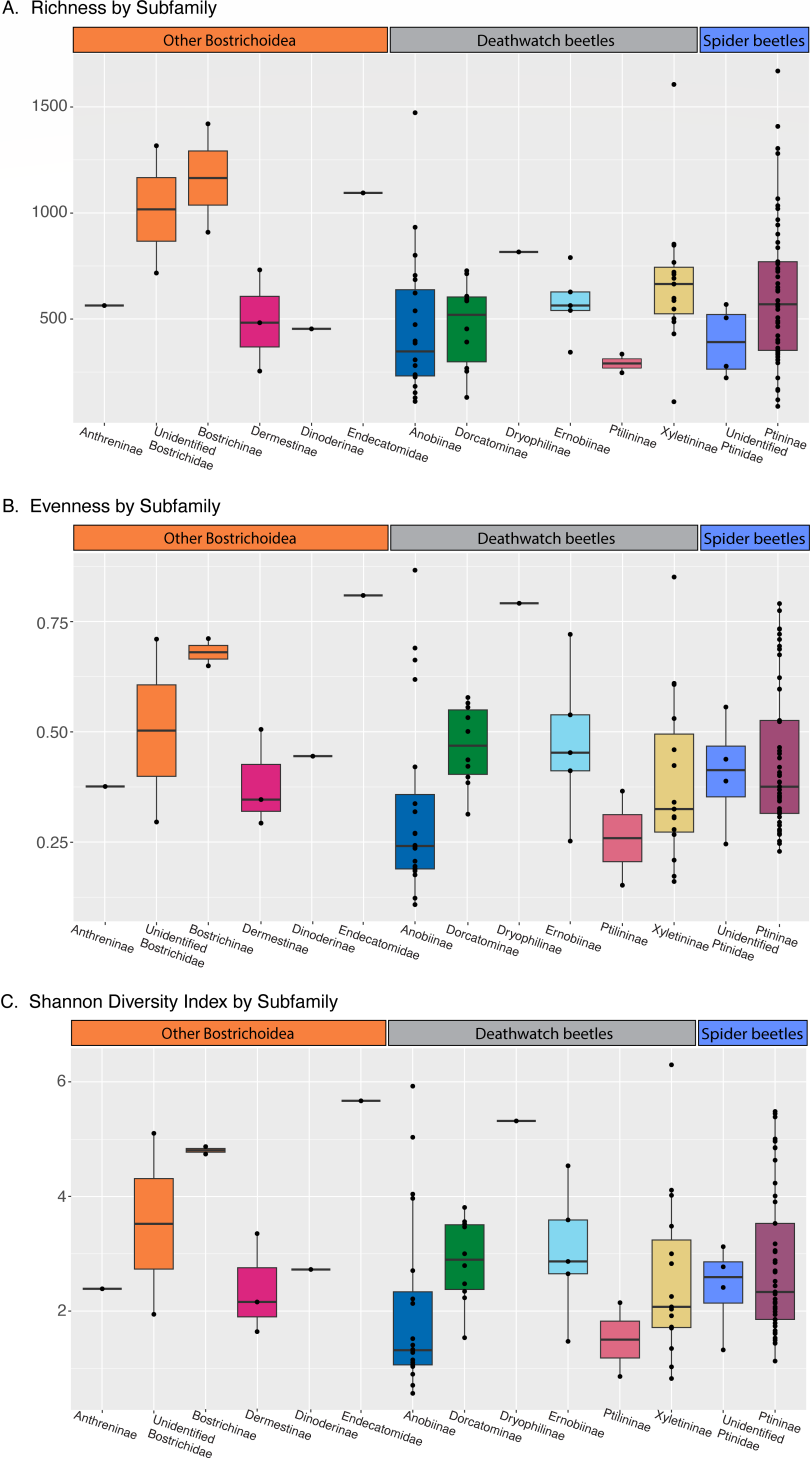
Bar plots of diversity metrics by subfamily (or family when subfamily was not identified). (A) Richness/alpha diversity, (B) evenness, and (C) Shannon diversity index.

**Fig 3:**
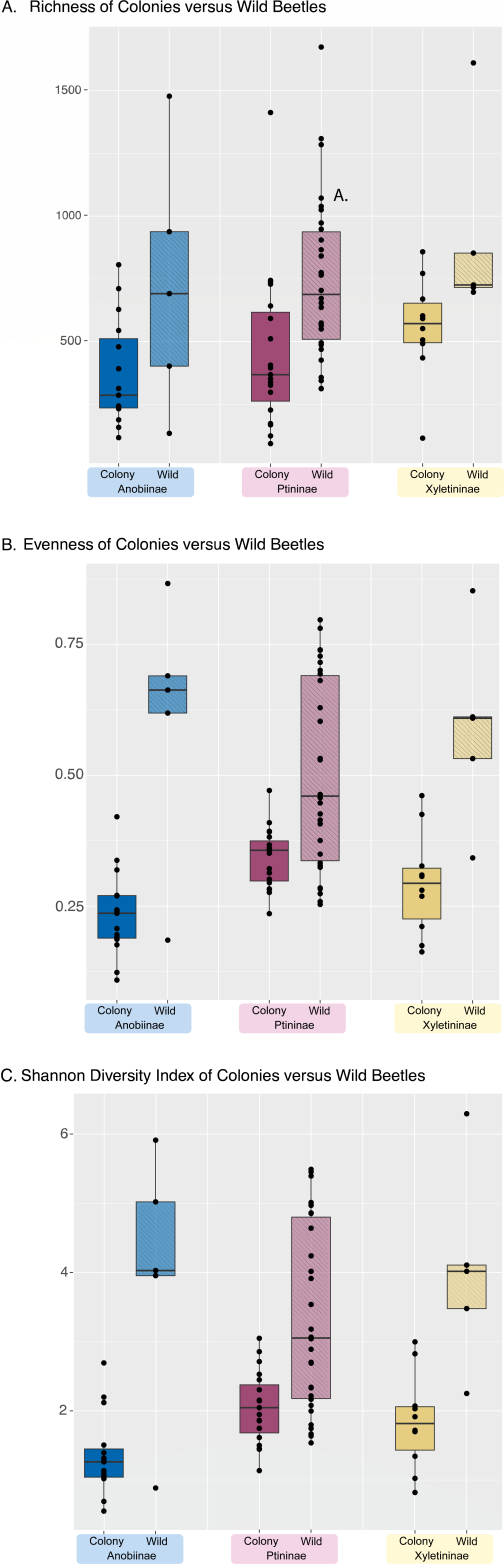
Bar plots of diversity metrics for three subfamilies with specimens that are either wild caught or from a colony. (A) Richness/alpha diversity, (B) evenness, and (C) Shannon diversity index.

Of the 116 specimens successfully sequenced, most were spider and deathwatch beetles, and the final data set also included five powderpost and four skin beetles. We were able to survey at least one species from eight of the nine Ptinidae subfamilies; however, sampling coverage varied based on specimen availability. We began by calculating overall diversity metrics for each family and subfamily of beetles. There was considerable variation in all diversity metrics among individuals. When grouped by subfamilies, powderpost beetles had the highest alpha diversity of all groups (Fig. 2A). There were no obvious trends with the average Shannon diversity indices and evenness for each subfamily (Fig. 2B and C), although some individuals like the single endecatomid and single dryophilinid had high metrics for each. Anobiinae, Ptininae, and Xyletininae all include beetles reared in colonies as well as wild-caught beetles. We hypothesized that some of the variation seen in these subfamilies could be explained by these environmental variables and re-ran the diversity statistics with these two groups differentiated (Fig. 3A through C). Potentially reflecting the greater dietary and environmental variation they experience, wild-caught beetles had higher values across all three diversity indices.

**Fig 4:**
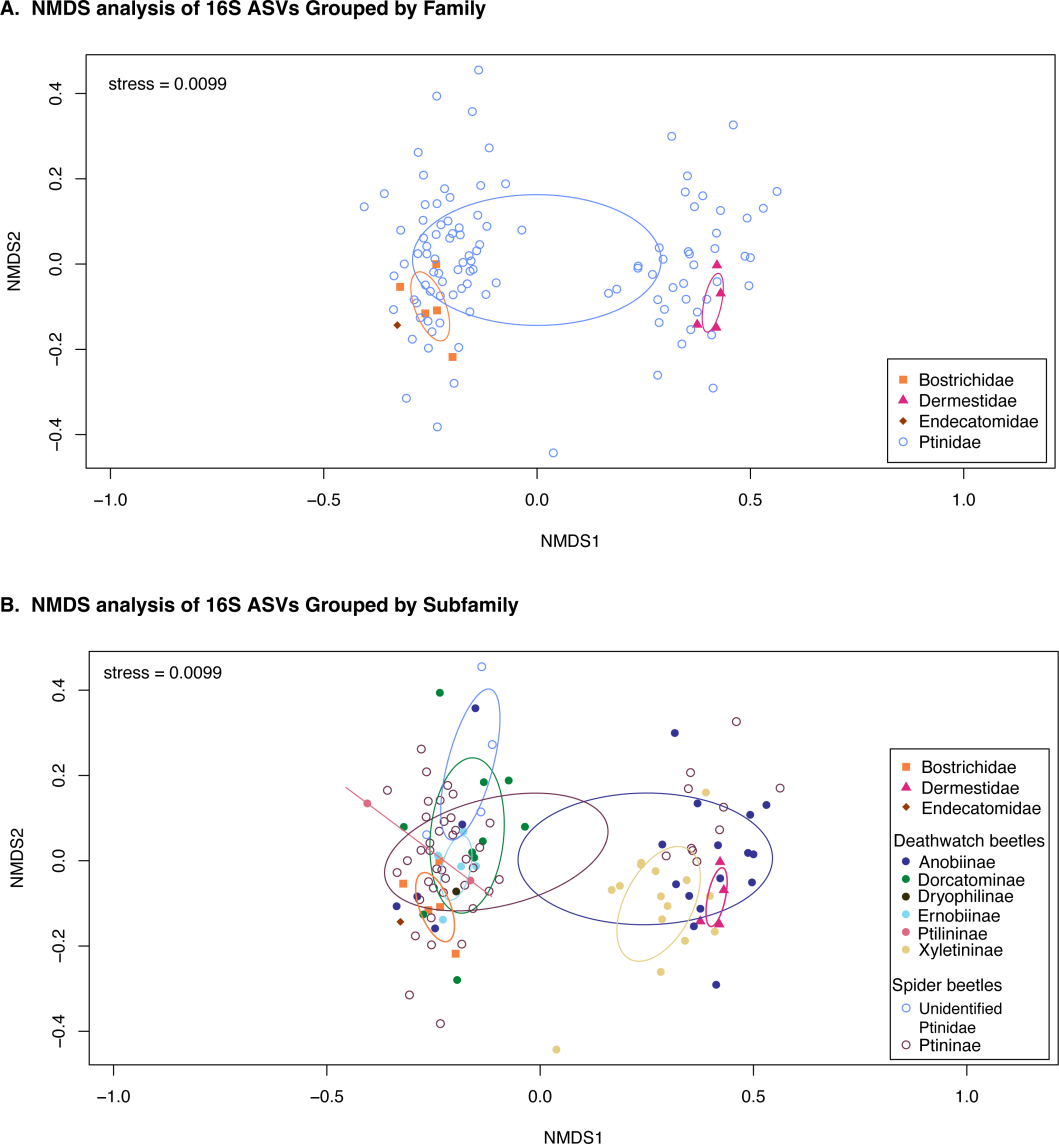
(A) NMDS plot comparing similarities between individuals in each bostrichid family. (B) NMDS plot comparing similarities between individuals based on families for outgroups (Bostrichidae, Dermestidae, and Endecatomidae) and subfamilies for ptinids. In cases where subfamily identification was not possible, the family is listed instead.

We then compared the overall microbiome compositions using NMDS plots (Fig. 4). There was significant overlap between all four families included in the data set, but bostrichids and dermestids formed clusters in composition space (Fig. 4A, stress = 0.0099). The endecatomid clustered near the bostrichids (Fig. 4A). Conversely, Ptinidae specimens seemed to fall into two distinct clusters. To address these clusters, we tried grouping the points by subfamily instead of family (Fig. 4B). In the left cluster are members of Dorcatominae and Ptilininae. In the right cluster is almost all Xyletininae. However, members of Ptininae and Anobiinae can be found in both clusters, albeit with different species on each side. This suggests that while some individuals have similarities in their bacterial communities, these commonalities may be driven by similar environmental conditions rather than indicating that taxonomic lineages consistently host specific bacterial communities. Much of the variation seen in ptinid beetles can also be explained by the large number of ptinid specimens relative to the other subfamilies. There are only four skin beetles, for instance, and three of those were sourced from the same colony.

We also looked at shared ASVs across all samples and only identified two that were present in every specimen: an unidentified Enterobacteriaceae and an *E. coli* sequence. The proportion of ASVs that correspond to these two sequences varies, but in most samples, they cover less than 1% of all ASVs identified, which leads us to believe that even if these reads are from contamination (either of the sequencing kit or other reagents during preparation), the contribution of contamination in this data set is minimal.

### Other Bostrichoidea (non-Ptinidae)

Previous studies have shown that bostrichid beetles have one or both of the bacterial endosymbionts *Bostrichidicola* and *Shikimatogenerans* (15, 16). In this data set, the *Lyctus* sp. had ASVs corresponding to both *Bostrichidicola* and *Shikimatogenerans*, as expected. *Prostephanus* sp. had a small number of *Bostrichidicola* sequences but was mostly dominated by *Shikimatogenerans*, possibly indicating that *Prostephanus* is in the evolutionary process of symbiont loss (*Bostrichidicola*), similar to what was found in *Rhyzopertha dominica* (16). Alternatively, this may simply indicate low yield from the initial DNA extraction or bias during the PCR. Similarly, the *Xylopertha* sp. and *Bostrichus capucinus* specimens did not have abundant ASVs for either endosymbiont when at least *Shikimatogenerans* was expected from previous surveys (16).

Neither *Bostrichidicola* nor *Shikimatogenerans* were present in dermestids. Instead, *D. maculatus* individuals were dominated by sequences of *Morganella* and an unidentified Enterobacteriaceae. The *Morganella* sequence most closely aligned to *M. morganii* and *M. psychrotolerans*, which are an opportunistic pathogen and a species associated with food poisoning, respectively (36, 37). All three *D*. *maculatus* individuals were sourced from a colony used to skeletonize mammal and bird specimens at the Museum of Southwestern Biology (MSB) at the University of New Mexico. Specimens being processed for accessioning to the MSB are sourced from diverse geographic locations. Because this *D. maculatus* colony subsists primarily on decomposing flesh with various origins, it is possible that either or both of these bacteria were acquired from their diet.

There was only one endecatomid in the data set, an unidentified *Endecatomus* species. There was no single ASV that dominated the *Endecatomus* sample. Most ASVs identified covered 5% of total ASVs or less. The only exception was *Sphingomonas*, which corresponded to 18% of all sequences identified in this sample. This was the only sample to have a significant amount (>2%) of ASVs corresponding to *Sphingomonas*.

### Spider beetles

**Fig 5:**
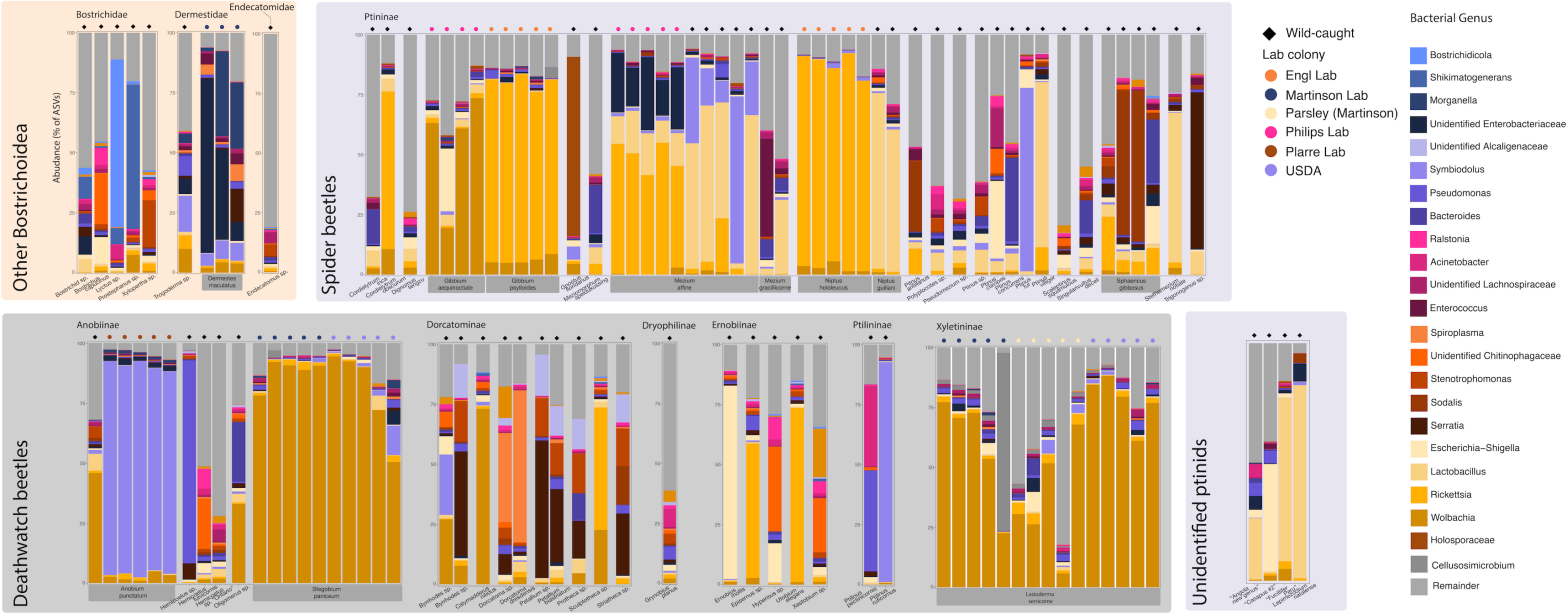
Stacked bar plot featuring the relative abundance of bacterial genera associated with each individual beetle. Any bacterial genera that had less than 3% of the relative abundance for at least one individual were grouped together under “remainder” for this plot. Bostrichids and dermestids are grouped by family, the remaining beetles are ptinids, which are grouped by subfamily, and then spaced by genera. The source of the beetle is indicated by a small symbol on top of each bar plot, where shape indicates wild versus lab colony, and color indicates specific population. Species with more than one individual in the data set are labeled with gray boxes beneath the bar plot.

*Ptininae*—Ptininae samples were sourced from a mix of laboratory populations and wild-caught specimens. Unsurprisingly, specimens from the same source had similar microbiomes (Fig. 5). While *Wolbachia* was present in both *Gibbium* species, *Rickettsia* was more common in Ptininae, with *Cordielytrum*, *Mezium*, *Niptus*, and *Sphaericus* all having sequences. *Mezium affine* and *Niptus guiliani* both had a significant portion of ASVs identified as *Lactobacillus rennini*, with *M. affine* from a lab population having over 10% of ASVs corresponding to *La. rennini* and wild *M. affine* and wild *N. guiliani* having over 50% of ASVs corresponding to *La. rennini*. Other species in Ptininae also had a *Lactobacillus*, but this was generally 5% of ASVs or less. Of the 616 *Lactobacillus* ASVs identified in the data set, 333 were found in *Ptininae*.

The two populations of *M. affine* each had unique microbiome compositions likely because they were maintained as colonies at different places with different diets. The *M. affine* lab colony had >50% of sequences corresponding to *Rickettsia*, whereas the wild-caught samples were more likely to have *Lactobacillus* or an unidentified Morganellaceae. *Sodalis*, a genus containing many insect endosymbionts found in taxonomically diverse hosts (38), was identified in *Pitnus antillus* and *Sphaericus gibbous. Gnostus floridanus* was dominated by *Holosporaceae*. Finally, an unidentified *Trigonogenius* had over 65% of its ASVs correspond to a single ASV identified as *Serratia*. While we cannot identify the species, many *Serratia* species are associated with insects primarily in the gut as pathogens or commensals, although the facultative symbiont *Serratia symbiotica* has been noted in aphids, where it confers a number of advantages, including protection from heat stress and parasitism (39, 40). Further investigation of the bacterial communities of *Trigonogenius* may be warranted.

There were four ptinids in the data set that were unable to be linked to a specific subfamily, which are labeled “Unidentified ptinids” in the figures. Three of the samples, “*Angola new genus*,” “*Fucillifer Peru*,” and *“Lepimezodium natalense*,” had over 20% of ASVs correspond to *Lactobacillus*; of those, *“Fucillifer Peru*” and “*Lepimezodium natalense*” had nearly 70% of ASVs correspond to *Lactobacillus*. Additionally, the “*Angola*” sample had nearly 30% of ASVs match *Comamonas*. Finally, the “Casapus #2” sample was mainly dominated by *Escherichia–Shigella* and *Massilia*.

### Deathwatch beetles

*Anobiinae*—The majority of samples in *Anobiinae* were sourced from lab colonies of *Anobium punctatum* or *Stegobium paniceum*, and we found that individuals within the same colony had very similar microbiomes (Fig. 3). While the wild *A. punctatum* primarily consisted of *Wolbachia* ASVs, the specimens from lab colonies had over 90% of ASVs correspond to an unidentified Morganellaceae endosymbiont (Fig. 5). When the sequence was entered into a BLAST search, it had a high percent identity to *Symbiodolus* (CP152425.1), a recently described arthropod symbiont (41).

There was little consistency in the microbiomes for the three *Hemicoelus specimens* (Fig. 5). For two of the specimens, *Hemicoelus fulvicornis* and *Hemicoelus* sp. *“Ontario*,” many of the same taxa were present, but only *H. fulvicornis* had over 20% of ASVs correspond to an unidentified Chitinophagaceae. The unidentified *Hemicoelus* sp*.* had over 80% of its ASVs correspond to a *Pseudomonas*. While *Pseudomonas* has been associated with bark beetle microbiomes, it has also been reported as an entomopathogen (42). Unfortunately, *Pseudomonas* species and strains cannot be differentiated based on the 16S rRNA sequences included in this study.

For the *Oligomerus* sp., a *Bacteroides* sequence was identified. However, this sequence does not appear to be related to *Bostrichidicola* or *Shikimatogenerans* and instead matched most closely to *Phocaeicola vulgatus*, which is associated with the human gut microbiome (43). *Oligomerus* sp*.* and *S. paniceum* additionally both had a large number of ASVs matching to *Wolbachia* (Fig. 5).

*Dorcatominae—Byrrhodes*, *Petallium*, *Protheca*, and *Striatheca* all had an ASV identified as a *Stenotrophomonas* comprisng 13–16% of all ASVs for those specimens (Fig. 5). One *Byrrohodes* specimen also had a high association (>25% of ASVs) with a different insect-associated Morganellaceae ASV than was seen in *A. punctatum*, although it had similar BLAST results (99.21% identity to CP152425.1), indicating they may be the same species of *Symbiodolus*. Both *Dorcatoma* specimens had over 25% of ASVs correspond to the same *Spiroplasma* ASV, which had a 100% identity on BLAST to numerous *Spiroplasma* arthropod endosymbionts, such as one for ticks (LC388762.1), *Drosophila melanogaster* (PP593989.1), and the pea aphid *Acyrthosiphon pisum* (KP710442.1). Either *Wolbachia* or *Rickettsia* were also found in *Byrrhodes*, *Calymadderus*, and *Sculptotheca. Striatheca* additionally had 16% of ASVs correspond to a *Sodalis*.

*Dryophilinae*—There was only one *Dryophilinae* specimen in the data set, a *Grynobius planus*. The proportion of each ASV was evenly distributed (Fig. 5), especially compared to the communities of other specimens, which can be seen in Fig. 2.

*Ernobiinae*—The dominant bacterial genera in Ernobiinae were *Escherichia–Shigella*, *Rickettsia*, and an unidentified Chitinophagaceae (Fig. 5).

*Ptilininae*—There were two specimens in this subfamily from the genus *Ptilinus*. ASVs in *Ptilinus pecticornis* were primarily split between *Pseudomonas* and *Acinetobacter. Ptilinus ruficornis* had over 90% of ASVs corresponding to a *Symbiodolus* as well, which may indicate an endosymbiotic infection in this particular specimen (Fig. 5).

*Xyletininae*—For this group, the only species represented is *Lasioderma serricorne*, which was sourced from three populations: a laboratory population maintained at the University of Mexico (UNM), a laboratory population from the USDA, and a population sourced from a commercially sold jar of dried parsley in Miami, Florida. All specimens were infected with *Wolbachia*. For the UNM and USDA colonies, over 50% of ASVs were *Wolbachia* sequences, with the exception of one specimen from the UNM colony that was infected with *Cellulosimicrobium*, corresponding to 74% of its total ASVs (Fig. 5). The “parsley” population was more diverse, as reported above, but did not have a dominant bacterial taxa other than *Wolbachia*.

### *Wolbachia* typing for *L. serricorne* and *S. paniceum*

*Wolbachia* infections have been previously reported in both *L. serricorne* and *S. paniceum*, and studies have utilized either *wsp* or MLST primers to genotype these endosymbionts (33, 35). We utilized the MLST primer set to survey our *L. serricorne* and *S. paniceum* populations, all of which harbored *Wolbachia* according to the 16S rRNA amplicon survey. These results varied in that individual genes were not amplified or had a mixed chromatogram in the Sanger results, which is why the “parsley” population was unable to be typed (Table 1). This indicated that there might be co-infection of multiple *Wolbachia* types in some populations. From the *Wolbachia* MLST genes successfully sequenced for *L. serricorne*, it appears that the USDA and UNM colonies each had unique *Wolbachia* strains, and the UNM colony *Wolbachia* closely matched that found in a Canadian population from reference 33. Among *S. paniceum*, the same *Wolbachia* strain was identified in the USDA and UNM colonies, but a unique strain was identified in an American population from reference 33. Overall, variations in *Wolbachia* infection and MLST types across populations suggest independent acquisitions of this common endosymbiont. It is unlikely that *Wolbachia* plays a mutualistic or facultative role in deathwatch beetle biology (as seen in blood-feeding insects such as ticks and mosquitoes); however, future experiments are required to test how eliminating *Wolbachia* affects host biology (44).

### Conclusion

Deathwatch and spider beetles have varied bacterial microbiomes with few commonalities. For this limited sampling set, we did not find a consistent bacterial endosymbiont common to all Bostrichoidea beetles, indicating that they lack an ancestrally shared obligate mutualist. However, this does not eliminate the possibility that some of the taxa that were consistently identified may be important to their hosts. The prevalent genera *Wolbachia* and *Rickettsia* likely represent facultative symbionts that either act as reproductive manipulators or provide context-dependent benefits to their hosts (e.g., protection from viruses). Additionally, amplicon sequencing is limited by the length of the sequences and quality of the DNA. It is possible that obligate symbionts exist, but we were unable to identify them or unable to amplify a sequence for them. This may also be why two of the powderpost beetles in this study did not have the expected *Bostrichidicola* or *Shikimatogenerans*. Along these lines, bacterial titer is generally not expected to correlate to the percentage of ASVs, so an unidentified symbiont could be present as a low percentage of ASVs we identified. Finally, sampling for many of these subfamilies was poor. The subfamily Eucradinae was not able to be included at all, and Dryophilinae and Ptilininae were represented by one and two species, respectively. It is possible that there may be more evidence toward an ancestral bacterial endosymbiont that could be found by a more thorough sampling of these groups.

One possible explanation for the lack of a common ancestral bacterial endosymbiont (or possibly the loss, or multiple losses, of one over time) is variations in dietary requirements between these families of beetles. For instance, while the currently recognized and described symbionts in powderpost (bacteria) and deathwatch beetles (fungal) provision hosts with nutrients used in development, they provide different metabolites: tyrosine and B-vitamins, respectively (15, 16, 24). This may suggest that the selective forces that created these symbiotic relationships occurred in each lineage independently following their divergence.

Obligate symbionts have been proposed as targets for new, specific pesticides, as many of the hosts of these obligate symbionts rely on them for the nutrients needed to complete development (45). However, other symbionts are also potential targets, such as *Wolbachia*, which has already been used to control the population of mosquitoes by inducing cytoplasmic incompatibility between treated and wild mosquitoes (46). In other cases, engineered *Wolbachia* strains have been used to partially vaccinate insects against diseases that they serve as a vector for, as attempted in mosquitoes against dengue and planthoppers against a rice virus (47). These techniques could potentially be used in the future to reduce beetle populations of the common pests*—L. serricorne* (the cigarette beetle) and *S. paniceum* (the drugstore beetle)—which both seem to have widespread *Wolbachia* infections, especially as phosphine resistance is becoming more common (48). Better understanding the microbiomes across the diversity of insects is critical for future control efforts and the development of new technologies to combat persistent, agriculturally and economically important pests.

## SUPPLEMENTAL MATERIAL

Table S1 spectrum.01981-24-s0001.xlsx Description of beetle specimens that includes sample code names for the supplemental data, source, location (when available), and taxonomic information.

Table S2 spectrum.01981-24-s0002.csv Counts table of microbiome data.

ASM does not own the copyrights to Supplemental Material that may be linked to, or accessed through, an article. The authors have granted ASM a non-exclusive, world-wide license to publish the Supplemental Material files. Please contact the corresponding author directly for reuse.
